# Synthesis, Characterization, and Toxicity Assessment of Superparamagnetic Iron Oxide Nanoparticles Coated with Antitumor Molecules

**DOI:** 10.1021/acsomega.4c09185

**Published:** 2025-04-24

**Authors:** Camila Chagas, Emerson B. da Silva, Beatriz da C. A. Alves, Glaucia L. da Veiga, Edimar C. Pereira, Paula Haddad, Maria Lúcia Schumacher, Tatiane Nassar Britos, Lídia M. Lima, Eliezer J. L. Barreiro, Fabio F. Ferreira, Fernando L. A. Fonseca

**Affiliations:** † Clinical Analysis Laboratory of the Centro Universitário FMABC, Av. Príncipe de Gales, 821, Bairro Vila Príncipe de Gales, Santo André, São Paulo 09060-650, Brazil; ‡ Chemistry Department, Federal University of São Paulo—Campus Diadema, Rua São Nicolau, 210, Centro, Diadema, São Paulo 09913-030, Brazil; § LASSBio, Institute of Biomedical Sciences, Federal University of Rio de Janeiro (UFRJ), Sala 35—Prédio Do Centro de Ciências da Saúde, Av. Carlos Chagas, 373—Bloco K, 2° Andar, Cidade Universitária, Ilha do Fundão, Rio de Janeiro, Rio de Janeiro 21941-902, Brazil; ∥ Graduate Program of Chemistry, Institute of Chemistry, Federal University of Rio de Janeiro (UFRJ), Av. Athos da Silveira Ramos, no 149, Bloco A—7° Andar, Centro de Tecnologia, Cidade Universitária, Rio de Janeiro, Rio de Janeiro 21941-909, Brazil; ⊥ Center for Natural and Human Sciences (CCNH), Federal University of ABC (UFABC), Santo André, São Paulo 09280-560, Brazil; # Nanomedicine Research Unit (NANOMED), Federal University of ABC (UFABC), Santo André, São Paulo 09280-560, Brazil

## Abstract

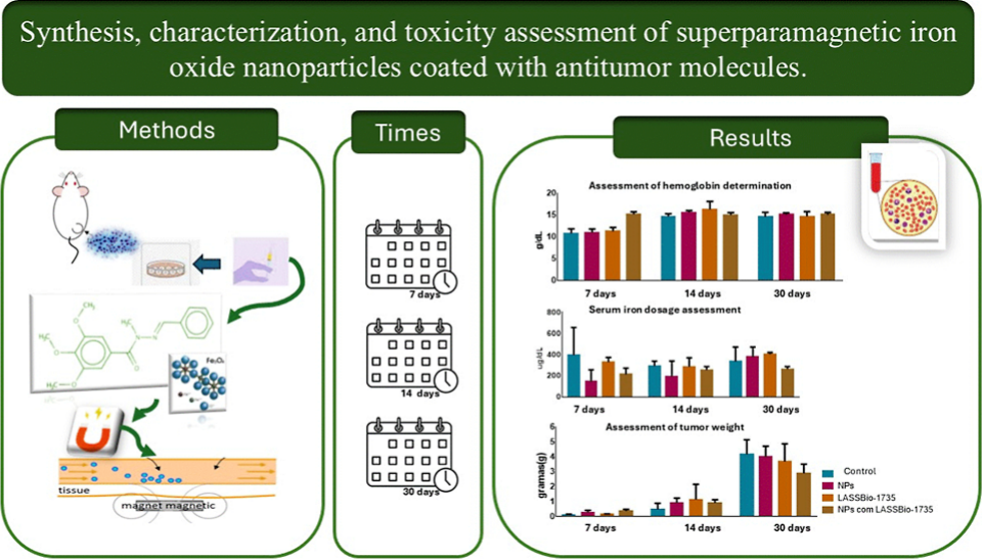

New definitions describe cancer as a condition where cells, transformed by natural selection, undergo uncontrolled proliferation. Currently, several available drugs for treating cancer present side effects and are nonselective, thus complicating patient treatment. To overcome such issues, alternative therapies using inorganic nanoparticles and parent compounds are becoming more attractive. Here, we demonstrate the use of biocompatible superparamagnetic iron oxide nanoparticles (SPIONs) coated with the LASSBio-1735 parent compound in BALB/c mice inoculated with the Ehrlich tumor. We first characterize the formed nanoparticles and confirm and quantify the anchoring of LASSBio-1735 on the surface of the SPIONs. Tests conducted after 7, 14, and 30 days show that no significant alterations were found in erythrocyte count analysis, hemoglobin and hematocrit determination, platelet and leukocyte counts, neutrophil/lymphocyte ratio, serum iron dosage, alanine aminotransferase, aspartate aminotransferase, serum creatinine, and urea determination, as well as analysis of the animals’ and tumor weights. This confirms that this new alternative can be considered in clinical trials to treat solid tumors.

## Introduction

The World Health Organization (WHO) estimates that approximately 704,000 new cancer cases will occur in Brazil annually during the triennium 2023–2025.^1^ Recent research links these data with changes in the population profile generated by globalization and changes in global demographics due to reductions in mortality and birth rates, leading to increased life expectancy and population aging.^2,3^

Cancer is the term for more than 100 diseases characterized by the uncontrolled growth of cells that invade tissues and organs, potentially spreading to other body regions.^4^ On the other hand, recent studies have revised this definition and considered cancer a disease of uncontrolled proliferation by transformed cells subject to evolution by natural selection.^5^ The most common types of cancer in men and women worldwide include breast cancer, lung cancer, colorectal cancer, prostate cancer, and nonmelanoma skin cancer. For women, the most common types are breast and colorectal cancers.^4^

Regarding the estimated annual mortality in 2023, the types of cancer that cause the most deaths among men in Brazil are prostate cancer, with 71,730 deaths, followed by colorectal cancer, with 21,730 deaths; trachea, bronchus, and lung cancer, with 18,020, and stomach cancer, with 13,340 deaths. Among women, breast cancer causes 73,610 annual deaths, followed by colorectal cancer, with 23,660; cervix cancer, with 17,010; and trachea, bronchus, and lung cancer, with 14,540 deaths.^4^

Breast cancer is the most common type among women worldwide, accounting for about 2.3 mi (24.5%) of new cases each year. Considered one of the most aggressive types in Brazil, excluding nonmelanoma cancer (118,000; 32.7%), breast cancer represents ∼74,000 (20.3%) new cases annually, followed by colorectal cancer, with 24,000 (6.5%) cases.^4^ Breast cancer also affects men, but it is rare, representing only 1% of total cases. Relatively rare in women under 35, its incidence progressively increases above this age, especially after 50. Statistics indicate an increase in incidence in both developed and developing countries, posing significant problems and requiring new strategies in healthcare.^4^

Several anticancer medications are currently available; however, their numerous side effects necessitate further research into alternative therapies. These innovative treatments can include various molecules and nanoparticles.^6^ The use of chemical compounds and metals has been reported since the earliest civilizations in Egypt, India, and China. The Chinese and Arabs used zinc for medicinal purposes, particularly for wound antisepsis, while the Egyptians used copper to sterilize water.^7,8^ During the Renaissance in Europe, mercury(I) chloride was employed as a diuretic, and significant efforts were made to understand the biological importance of iron in the diet. Based on this knowledge, researchers began developing chemical compounds and particles with biological activity.^9−12^

In recent decades, the development of nanoparticulate materials has received much attention due to their numerous applications, ranging from catalysis to biomedicine.^13−17^ Among nanometric structures, superparamagnetic iron oxide nanoparticles (SPIONs), such as magnetite (Fe_3_O_4_) and maghemite (γ-Fe_2_O_3_), are of great interest due to their unique properties, such as superparamagnetism and biocompatibility. Being biocompatible, they allow the incorporation of drugs onto their functionalized surfaces, which can be directed to their site of action by applying an external magnetic field. This reduces side effects, lowers the risk of toxicity, decreases the circulation time in the body, and allows for adequate control of the dose, site of action, and elimination time.^16,18−21^ Given this, there has been an increased search for new drugs, including bioactive molecules such as combretastatin A4 (CA-4). This structurally simple molecule exhibits potent cytotoxic activity in in vivo and in vitro assays by inhibiting microtubule polymerization.^22^

In this context, at the Laboratory for Evaluation and Synthesis of Bioactive Substances (LASSBio) at the Federal University of Rio de Janeiro (UFRJ), a series of new CA-4 analogs were synthesized, with the LASSBio-1586 compound standing out.^23^ Subsequently, LASSBio-1735 was synthesized, showing promising results regarding its cytotoxic effects against tumor cell lines such as HL-60 (human leukemia), SF295 (human glioblastoma), MDA-MB435 (melanoma), and HCT-8 (ileocecal carcinoma—colon).^24^

The Ehrlich tumor,^25^ described in the early 20th century by scientist Paul Ehrlich in 1906, is an experimental transplantable neoplasm of malignant epithelial origin, corresponding to mammary adenocarcinoma in female mice. It is covered by a pseudo capsule composed of pleomorphic cells with abundant cytoplasm, which may contain vacuoles. Additionally, large multinucleated cells and mitosis appear frequently in this tumor, with extensive necrosis resulting from the death of neoplastic cells.^26,27^ The Ehrlich tumor is commonly used to study alternative treatments for breast cancer, as it can develop in various strains of this animal species in both ascitic and solid forms.^25,28−30^ When subcutaneously implanted, it is obtained in the solid form. When tumor cells are injected into mice’s peritoneal cavity, as Loewenthal and Jahn proposed,^31^ the tumor develops in its ascitic form, referred to as Ehrlich ascites carcinoma (EAC).^32^

The choice to analyze the biological applications of SPIONS associated with molecules that have shown antitumor activity sparked interest due to the possibility of synergistically increasing the available drug options based on the results observed with biomedical applications of superparamagnetic iron oxide nanoparticles and anticancer molecules.^14−17,24^

Most available antineoplastic agents have undesirable side effects and lack selectivity, hindering patient treatment. In this regard, advances in research in the inorganic and organic medicinal chemistry fields are promising, offering various possibilities. Therefore, we have decided to focus on analyzing new molecules and using SPIONs to observe these systems’ toxicity and biological properties against the Ehrlich tumor.

Thus, the present study aims to obtain and characterize SPIONs and evaluate their conjugation with LASSBio-1735 in BALB/c mice inoculated with the Ehrlich tumor regarding toxicity among the different treated groups. We focus on assessing the effects of SPIONs and LASSBio-1735, both individually and anchored, on myelotoxic and hepatotoxic activities and evaluating if they possess antitumor activity. We analyze animal well-being following the study model developed by the National Centre for Replacement, Refinement & Reduction of Animals in Research.^33^

## Methodology

All experimental procedures described in this study were approved by the Animal Experimentation Ethics Committee of the University Center FMABC under Law 11.794/2008 (Arouca Law), protocol number 012/2018.

### Synthesis of Superparamagnetic Iron Oxide Nanoparticles

SPIONs were synthesized through the coprecipitation method using FeCl_3_·6H_2_O (0.5 mol L^–1^) and FeCl_2_·4H_2_O (1 mol L^–1^) solutions in an acidic environment. This mixture was vigorously stirred at room temperature while an ammonium hydroxide (NH_4_OH) solution (0.7 mol L^–1^) was slowly added until a black precipitate formed.^34^ After the drip, the precipitate was magnetically decanted and washed approximately 5 times with ethanol, then dried under vacuum at room temperature for 2 days.

### Adsorption of l-Cysteine on the Surface of Fe_3_O_4_ Nanoparticles

SPIONs (∼200.0 mg) were suspended in 5.0 mL of water, and simultaneously, 2.0 g of l-cysteine was dissolved in an equal volume of water. The SPION suspension and the l-cysteine solution were combined and vigorously stirred for 14 h. This process yielded a black powder, which was magnetically separated and washed five times with ethanol. The result was the production of water-stable, thiol-containing nanoparticles (Cys-SPIONs).^21^

### Incorporation of the LASSBio-1735 onto the Surface of the Cys-SPIONs

For the synthesis, 0.02 g of l-cysteine-SPIONs were combined with 0.10 g of LASSBio-1735 in an ethanol solvent medium, maintaining a mass ratio of 1:5 (Cys-SPIONs to LASSBio-1735). The dispersion was vigorously stirred at room temperature for 24 h to ensure homogeneity. Following this, the resultant solid was isolated using a magnetic decantation process and subjected to repeated washing, approximately 5 times, with ultrapure water to eliminate any residual impurities. Finally, the purified product was dried under vacuum at room temperature for 5 days to ensure optimal dryness and stability, preparing it for subsequent characterization and application assessment.

### X-ray Powder Diffraction (XRPD)

X-ray powder diffraction data were collected on a STADI-P (Stoe, Darmstadt, Germany) powder diffractometer operating at 40 kV and 40 mA, in transmission geometry, using Cu*K*α_1_ radiation (λ = 1.54056 Å). The diffracted intensities were recorded by a Mythen 1 K (Dectris, Baden, Switzerland) linear detector from 4.000° to 60.685°, in steps of 0.015° and a counting time of 120 s at each 1.05°.

### Fourier Transform Infrared (FT-IR) Spectroscopy

FTIR spectra were obtained using the Agilent Cary 630 (Agilent, Santa Clara, CA, USA) spectrometer with a diamond crystal ATR accessory. The measurements were carried out from 400 to 4000 cm^–1^ with a resolution of 4 cm^–1^.

### Quantification of Free Thiol Groups on the Surface of SPIONs

The thiol groups (-SH) on the surface of the nanoparticles were quantified by titration with 5′,5′-ditiobis(2-nitrobenzoic acid) (DTNB) using the ultraviolet–visible spectrophotometry technique. The free –SH reacts with DTNB to form 5-mercapto-2-nitrobenzoic acid (TNB2−) with a characteristic absorption band at 412 nm (ε = 11,400 M^–1^ cm^–1^). Briefly, 10 mg of NPs was dispersed in 1.5 mL of TBE buffer (TRIS-borate EDTA) and added to 200 μL of DTNB (5.07 mmol L–1) in TBE buffer (pH 8.3) with 1 mmol L^–1^ of ethylenediaminetetraacetic acid (EDTA). After 5 min of incubation, the suspensions were centrifuged. The supernatant was placed in a quartz cuvette, and the absorption band at 412 nm was measured using a UV–vis spectrophotometer (Agilent, model 8553). The experiments were carried out in triplicate, and the standard deviation was estimated.

### Magnetization Curves

Magnetization measurements were performed using a superconducting quantum interference device (SQUID) magnetometer, model MPMS XL7, from Quantum Design (San Diego, CA, USA) at the Experimental Multiuser Center (CEM) of the Federal University of ABC (UFABC). The measured temperature was 300 K, and the samples were analyzed as dried powder, which was pressed and conditioned in cylindrical holders of Lucite.

### Dynamic Light Scattering and Zeta Potential

Dynamic light scattering measurements were performed using a compact ALV/CGS-3 goniometer system consisting of a 22 mW linearly polarized He–Ne laser operating at a wavelength of λ = 633 nm, an ALV 7004 digital correlator and a pair of avalanche photodiodes operating in the pseudocross correlation mode. Autocorrelation functions were obtained in the 90° angle region and adjusted using the cumulative method. The Zeta Potential of the synthesized samples was measured on the Zetasizer Nano ZS, Malvern Instruments, using the electrophoretic light scattering (ELS) technique. The suspensions were prepared for both analyses by dispersing approximately 10 mg of the sample in 20 mL of water using ultrasound at a consistent temperature of 25 ± 1 °C.

### Transmission Electron Microscopy (TEM)

The morphology and size distribution of the superparamagnetic NPs were identified using a Talos F200X G2 Transmission Electron Microscope with cold gun field-effect emission (FEG-X), scanning module (STEM), and atomic resolution capability (HRTEM). The samples were suspended in ethanol, and then a drop of the supernatant dispersion was deposited on an amorphous carbon film supported by a copper grid. The size distribution and average diameter were calculated from 250 nanoparticles.

### Chemotherapeutic

To complement the experimental design, we used a drug with a well-known action, such as doxorubicin, commercially known as Adriamycin or hydroxydaunorubicin. The cytotoxic properties of doxorubicin on malignant cells and the toxic effects on various organs appear to be related to its intercalation into nucleotide bases and its ability to bind to the lipid cell membrane. Doxorubicin was administered to mice via intraperitoneal injection (IP) at 2.5 mg kg^–1^ once every 5 days.

### Animals

This experimental study consisted of male albino mice, BALB/c strain, with an average weight of 30 ± 5 g, obtained from the Animal Facility of the ABC Medical School. Each treatment group consisted of 6 animals. During the experiment, the animals were housed in polypropylene cages (49 × 34 × 16 cm), lined with wood shavings changed twice a week, and maintained under a 12 h light/dark cycle with controlled ventilation (20 air changes per hour), temperature, and relative humidity, and provided with filtered water and Nuvilab CR-1 (Nuvital) pellet diet ad libitum.

The assessment was conducted over 3 different periods: the first for 7 days, followed by 14 days, and finally 30 days.

All animals were inoculated with tumor cells.

The animals were divided into 8 groups:

- Group 1 (*n* = 6): Animals received 0.1 mL of deionized water.
- Group 2 (*n* = 6): Animals received subcutaneous injection of 0.1 mg mL^–1^ nanoparticle solution (0.1 mL).
- Group 3 (*n* = 6): Animals received subcutaneous injection of 0.1 mg mL^–1^ LASSBio-1735.
- Group 4 (*n* = 6): Animals received subcutaneous implantation of a magnet and 0.1 mL of deionized water.
- Group 5 (*n* = 6): Animals received subcutaneous implantation of a magnet and subcutaneous injection of 0.1 mg mL^–1^ nanoparticle solution (0.1 mL).
- Group 6 (*n* = 6): Animals received subcutaneous implantation of a magnet, subcutaneous injection of 0.1 mg mL^–1^ nanoparticle solution (0.1 mL), and 0.025 μM LASSBio-1735.
- Group 7 (*n* = 6): Animals received a subcutaneous injection of nanoparticle solution 0.1 mg mL^–1^ (0.1 mL) combined with 0.025 μM LASSBio-1735.

### Inoculation of Ehrlich Tumor

To obtain the solid tumor, tumor cells obtained from the ascitic fluid of mice with ascitic Ehrlich tumor with 7 days of evolution from the strain originating from the FMABC animal facility were used. Trichotomy was performed on the animals using a hair clipper on the dorsal region of the mouse, and the cell suspension was injected via a 24-gauge needle at a concentration of 2 × 10^5^ cells mL^–1^ (0.1 mL) into the lateral area of the dorsum of each animal. After 14 days of inoculation, we started the count as the experiment’s zero time.

### Magnet Implantation

As demonstrated in the image below, neodymium magnets with dimensions of 3 mm × 2.5 mm, disk-shaped, and N32 grade were used. Animals in groups 5, 6, and 7 were trichotomized and then anesthetized with 2% isoflurane for magnet implantation in the dorsal region of the animals. A small incision was made in the dorsal region of the mouse, then the magnet was implanted, and the skin was glued with cyanoacrylate glue. As the procedure is virtually painless, the animals did not receive analgesics in the immediate postoperative period. Treatments were performed immediately after magnet implantation.

### Analysis of Animal Welfare

We created a table in which scores were assigned to each factor, such as the presence of ulcers, evaluation of fur, movements, posture, tail, eyes, ears, and whiskers. A score of 0 (zero) was assigned to an ideal situation, i.e., adequate animal welfare, and a score of 3 (three) would be assigned to a problem of animal suffering. Thus, the higher the score, the worse the animal’s condition. All these parameters were analyzed daily, with scores assigned to each parameter daily during the experiment. In cases where the total score exceeded 19, a humane end point would be determined, and euthanasia of the animal would be performed by an overdose of anesthetic with the anesthetics mentioned above.

### Euthanasia and Sample Collection

After the study periods ended, the animals were euthanized with sodium thiopental 100 mg kg^–1^ intraperitoneally. Blood was collected by puncturing the caudal vena cava and stored in pediatric tubes with EDTA for hemograms and to measure hepatic enzymes and serum iron levels at the Clinical Analysis Laboratory of FMABC.

### Biochemical and Hematological Analysis

The determination of alanine aminotransferase (ALT), aspartate aminotransferase (AST), serum iron, serum creatinine, and urea in plasma was performed by the kinetic-UV method using the ADVIA 1200 Siemens automation equipment, using InVitro reagents, following good practices in clinical analysis. The hematological evaluation was performed by flow cytometry, using a Sysmex XN-2000 Hematology System (Melville, NY, USA), which included erythrocyte count, leukocyte count, platelet count, and microscopic analysis of blood smears to observe possible leukocyte and erythrocyte alterations, following good practices in a clinical study.

### Results Analysis

Absolute and relative values were used for qualitative variables. Median, 95% confidence interval, and percentiles 25 and 75 were used to express non-normal quantitative data (Shapiro–Wilk <0.05). For data representing normality (Shapiro–Wilk >0.05), mean, standard deviation, minimum and maximum values were used. Chi-square, Kruskal–Wallis, and One-way ANOVA tests were performed to test differences between and within groups for the recorded parameters and postanalysis Sidak’s multiple comparisons tests. A confidence level of 95% was used for all analyses. The statistical program used was GraphPad Prism version 8.0.^24^

## Results and Discussion

**Figure 1:**
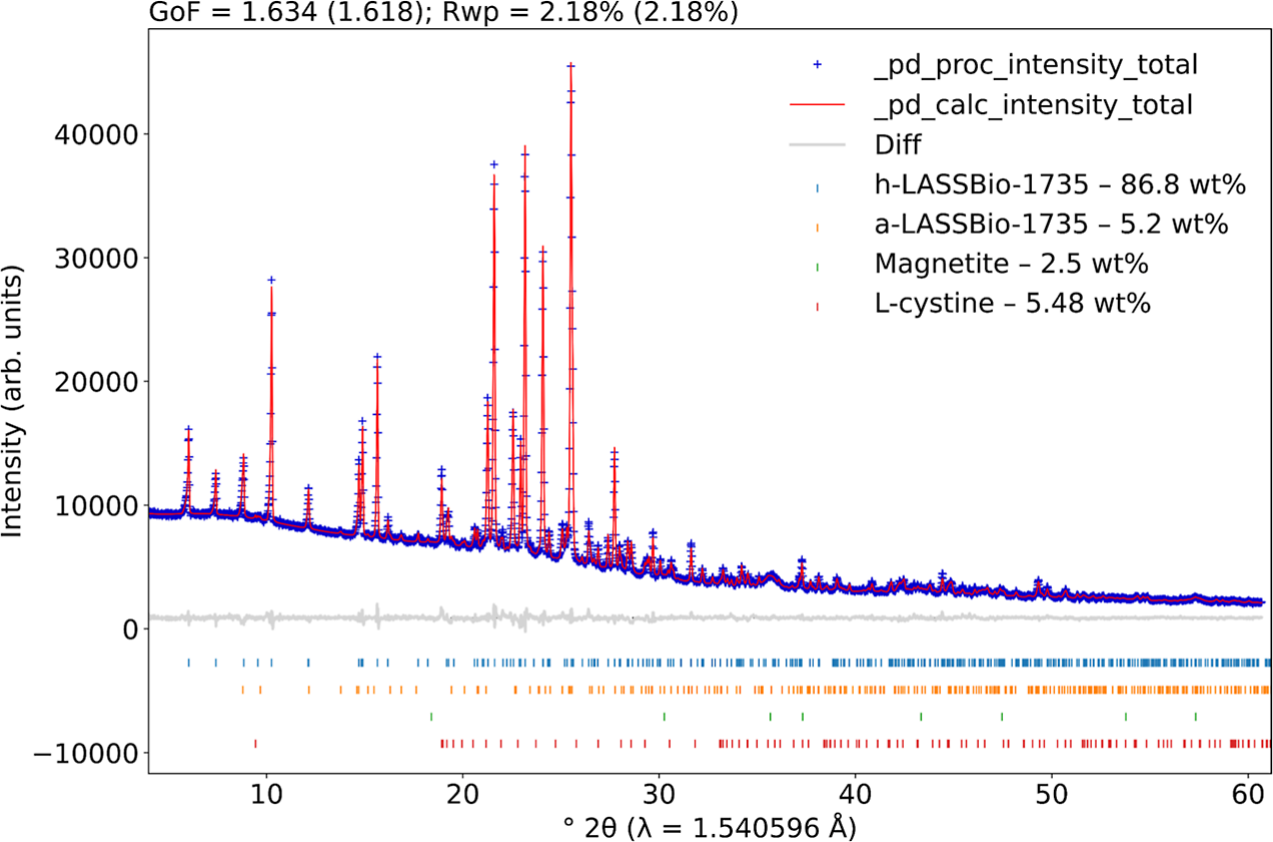
Rietveld plot of the SPIONs-Cys-LASSBio-1735 sample. The blue crosses indicate the observed pattern, while the red line displays the calculated one. The gray line represents the difference between the observed and calculated pattern, and the vertical lines at the bottom indicate the Bragg reflections of the observed phases.

A quantitative phase analysis^35,36^ using X-ray powder diffraction data and the Rietveld method^37^ was carried out as an initial screening of the structural characterization of the synthesized nanoparticles and to infer the successful attachment of the LASSBio-1735 molecule to the surface of the SPIONs. We used the crystal structures in the literature to describe iron oxide,^38^ LASSBio-1735,^24^ and l-cystine^39^ contributions. Although we have used l-cysteine in the synthesis procedure, we could infer that it transforms to its dimer (l-cystine) in the solid state. On the other hand, in solution, it goes back to l-cysteine.^21^Figure 1 shows the Rietveld plot of the SPIONs-Cys-LASSBio-1735 sample, indicating that we obtained 86.8(4) wt % of anhydrous LASSBio-1735, 5.2(2) wt % of hydrated LASSBio-1735, 2.5(3) wt % of magnetite, and 5.5(1) wt % of l-cystine. The software *pdCIFplotter*(40) was used to generate Figure 1.

To determine the chemical composition of both as-synthesized (Fe_3_O_4_) SPIONs-Cys and SPIONs-Cys-LASSBio-1735, we conducted Fourier-transform infrared spectroscopy (FTIR) analysis.

**Figure 2:**
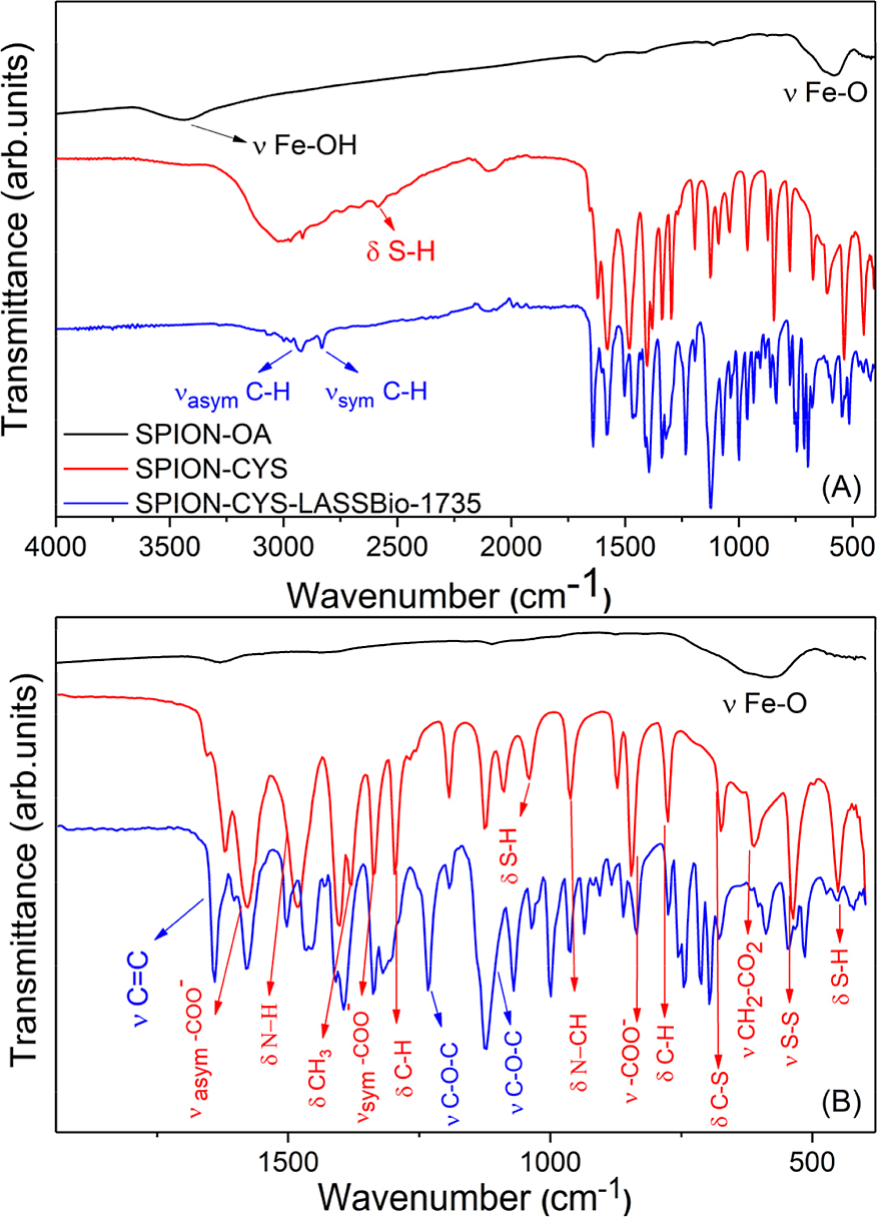
(A) FTIR to samples SPIONs; SPIONs-Cys and SPIONs-Cys-LASSBio 1735. (B) Enlarged region (1900–400 cm^–1^) displaying some of the most characteristic bands of l-cys (red) and LASSBio-1735 (blue).

Figure 2 presents the main absorption bands observed in the spectra of these samples. As detailed in previous studies, the characteristic bands of the iron oxide core are evident at 3460 cm^–1^ (νFeO–H) and 579 cm^–1^ (νFe–O).^41^ The presence of l-cysteine is confirmed by bands around 1654, 1639, 1589, 856, 825, and 760 cm^–1^, which are linked to the asymmetric bending of the NH_3_ group.^42,43^Table 1 summarizes the vibrational modes of l-Cys along with their respective wavenumbers.

**Table 1:** Assignment of Bands for the Vibrational Modes of l-Cysteine

wavenumber (cm^–1^)	vibrational modes
3175	ν–NH_2_
2957	ν_asym_ C–H aliphatic
2780	Ν_sym_ C–H aliphatic
2550	δ (S–H)
1572	ν_asym_–COO^–^
1520	δ (N–H)
1422	δ CH_2_
1383	δ CH_3_
1348	ν_sym_ –COO^–^
1284	C–H (bending)
1136	δ NH_3_
1054	S–H (in plane bending)
940	ν N–CH
860	CO_2_ (rocking)
800	CO_2_ (scissoring)
772	δ C–H
670	δ C–S
636	ν CH–CO_2_
450	δ S–H

In the spectra shown in Figure 2, shifts associated with cysteine and iron oxide bands are observed, indicating interactions between these molecules. For example, the band at 580 cm^–1^, characteristic of the Fe–O bond in SPIONs, shifts to 600 cm^–1^ in the spectrum of functionalized SPIONs. This shift to higher frequencies suggests that the Fe–O bond is stronger than the Fe-Cys bond, indicating adsorption between Fe and l-Cys rather than the formation of a chemical bond. This adsorption between iron and cysteine is further evidenced by changes in the shape of the bands at 1580 and 1500 cm^–1^, attributed to the vibrations of the carboxylate ion (ν_asym_ –COO^–^) and the nitrogen–hydrogen bond (δ N–H), respectively. The carboxylate and amino groups have free electrons, which can interact with the iron oxide surface, allowing cysteine to bind to the metal. Variations in the molecules’ dipole moments, likely due to differing electronic densities, may lead to adsorption via van der Waals interactions.

However, cysteine in the solid state can form its dimer, l-cystine, through disulfide bridges resulting from thiol oxidation. The thiol group has a characteristic band at 2550 cm^–1^ in the l-Cys spectrum. However, this band is absent in the spectra of SPIONs with cysteine (red spectrum), and additional bands appear that cannot be attributed to cysteine alone. This suggests a likely mixture of cysteine and cystine, which was later confirmed by XRD results.

We also observed bands from 3040 to 1240 cm^–1^, corresponding to CH groups’ stretching and bending vibrations.^44^ Additionally, the band at 530 cm^–1^ indicates the presence of the S–S bond.

FTIR spectra of SPIONs, SPIONs-Cys, and SPIONs-Cys-LASSBio-1735 reveal distinct differences, especially in the latter. Specifically, the bands ranging from 1275 to 1020 cm^–1^, associated with the C–O bond of the ether group in the LASSBio-173 molecule, and the band at 1640 cm^–1^ referring to the alkene group of the aromatic ring, illustrate the chemical modifications introduced by the binding of LASSBio-1735.

It is also worth noting that the SH groups were quantified by titration using 5′,5′-dithiobis(2-nitrobenzoic acid) (DTNB), with Ultraviolet–visible Spectrophotometry (UV–vis). In an exchange reaction, DTNB, containing a highly oxidizing disulfide bond, is stoichiometrically reduced by free thiol groups (–SH), forming a mixed disulfide and releasing the TNB^–^ anion, which has a p*K*_a_ of 4.5. For each thiol oxidized in this reaction, one TNB^–^ is released. A yield of 1.15 mmolg^–1^ of thiols was obtained on the surface of the SPIONs.

**Figure 3:**
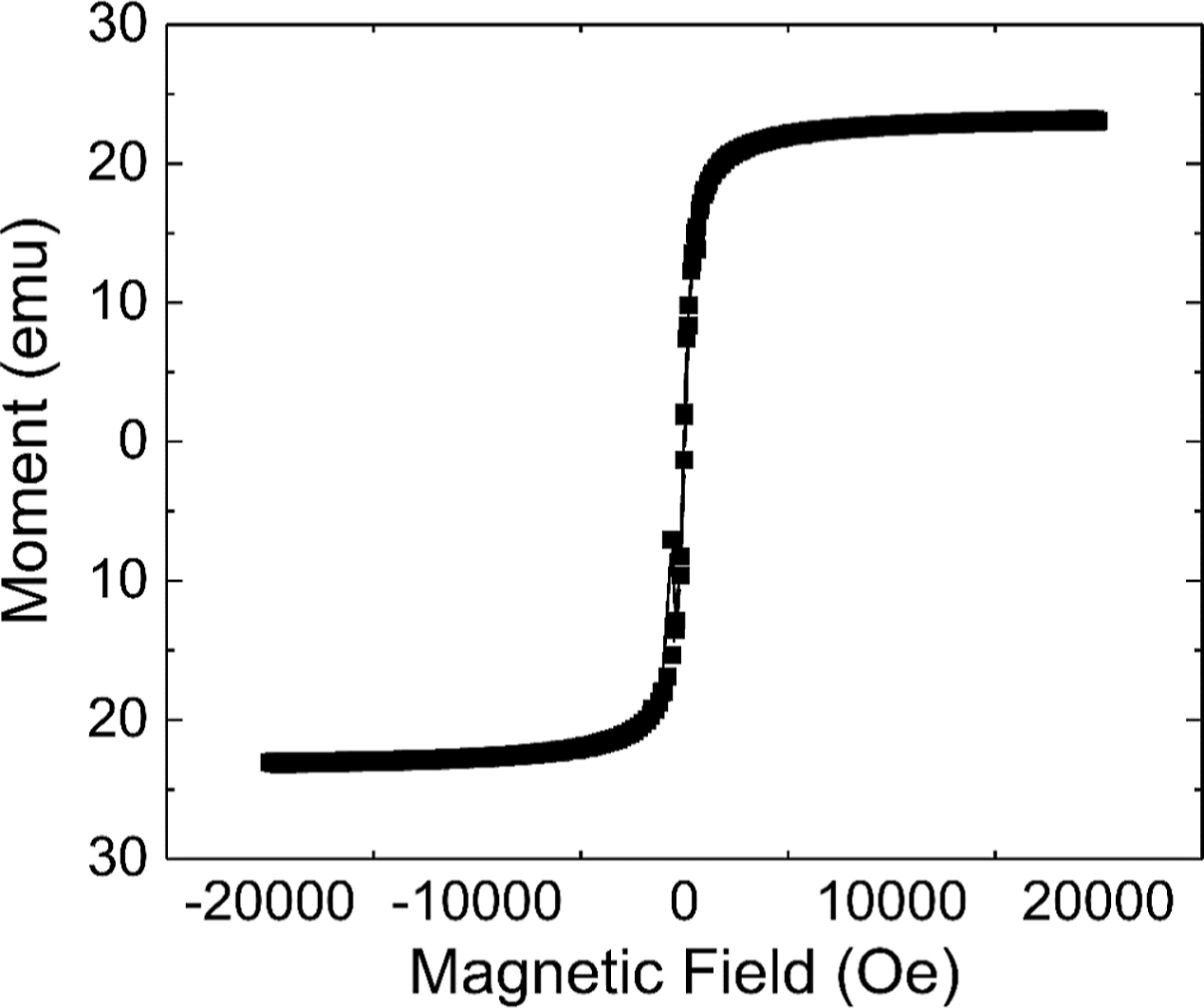
Magnetization curve for SPIONs-Cys-LASSBio-1735. The hydrodynamic sizes of SPIONs-Cys and SPIONs-Cys-LASSBio-1735 nanoparticles were found to be approximately (80.0 ± 7.1) nm and (129.0 ± 5.0) nm, respectively. This increase in size (from 80 to 129 nm) is associated with the anchoring of LASSBio to the SPIONs surface. The PDI values increased from 0.20 ± 0.07 for SPIONs-Cys to 0.49 ± 0.05 for SPIONs-Cys-LASSBio-1735. Finally, regarding zeta potential values, we observe an increase from (−30.00 ± 0.35) mV for SPIONs-Cys to (−32.00 ± 0.50) mV for SPIONs-Cys-LASSBio-1735. A zeta potential of −30 mV indicates excellent colloidal stability and reflects the surface charge of the nanoparticles.

Figure 3 presents the magnetization curve of SPIONs-Cys-LASSBio-1735 obtained under isothermal magnetic conditions at room temperature. The hysteresis loop indicates superparamagnetic behavior for this sample. Additionally, we observed that the residual magnetization and the coercive force were zero for the sample. The saturation magnetization (Ms) was approximately 20 emu g^–1^. This value is lower than the saturation magnetization of around 80 emu g^–1^ reported for Fe_3_O_4_ in our previous studies.^21,34^ The decrease in Ms can be attributed to the addition of two nonmagnetic layers, l-cystine and LASSBio-1735, on the surface of the SPIONs. This modification suggests that SPIONs-Cys-LASSBio-1735 nanoparticles can be manipulated by an external magnetic field and directed toward a specific target.

**Figure 4:**
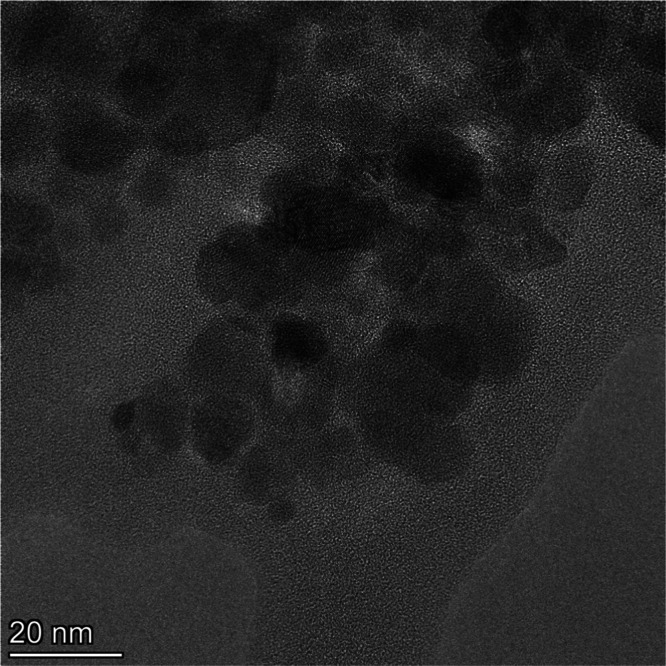
TEM image of SPIONs-Cys-LASSBio-1735 For cytotoxicity analysis, we chose commonly used standards for determining hematocrit, hemoglobin, platelets, red blood cells, and white blood cells.

Figure 4 depicts the TEM image of SPIONs-Cys-LASSBio-1735. The nanoparticles are almost spherical but show aggregation due to surface interactions from intermolecular forces. It is worth observing the crystalline planes related to magnetite. The nanoparticles are around 15 nm in size.

**Figure 5:**
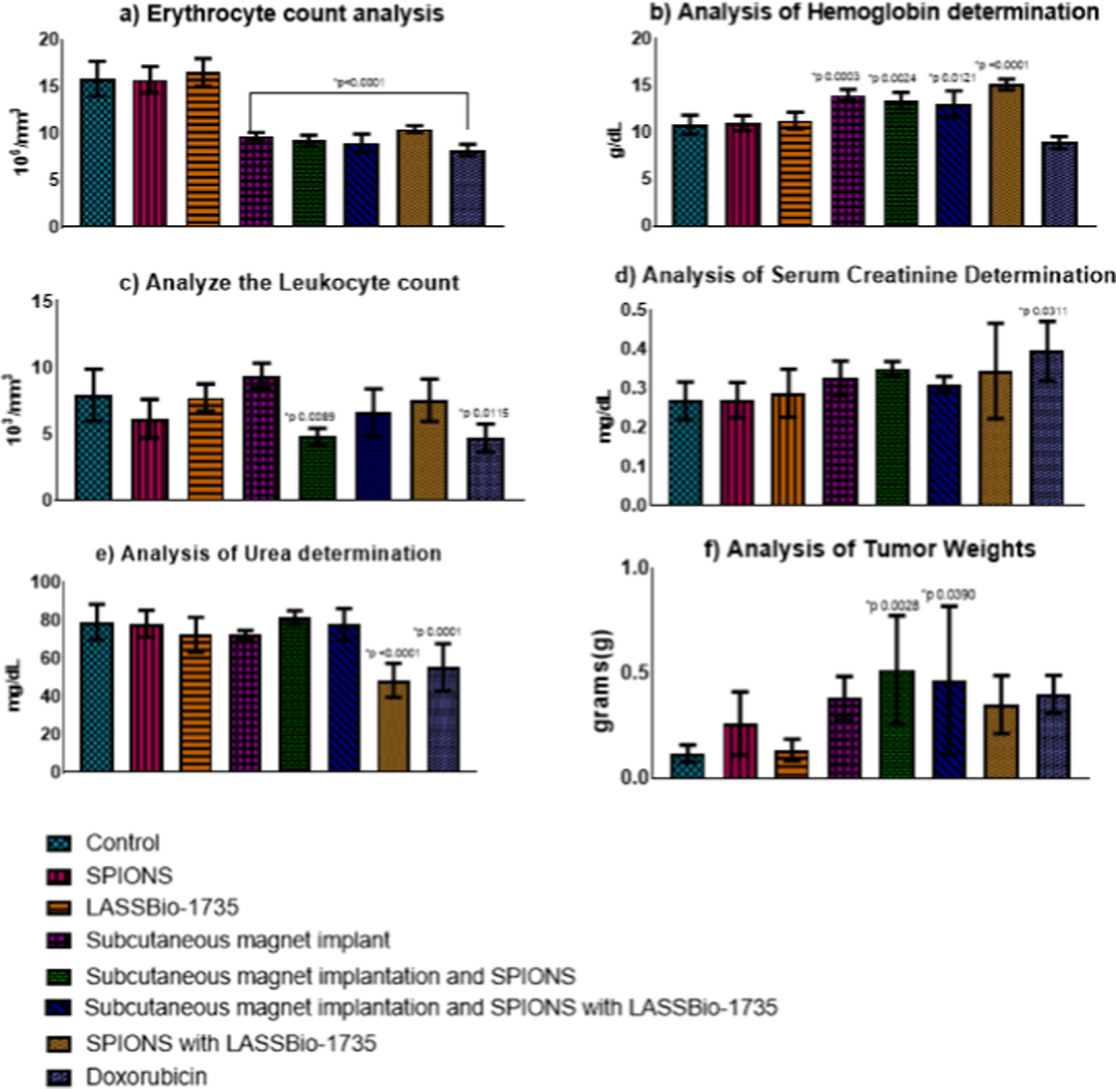
Myelotoxicity analysis, with evaluation of red blood cells, hemoglobin, leukocytes, urea, creatinine, and tumor size, over 7 days, *p < 0.05.

In the evaluation of erythrocytes, a significant difference (*p* < 0.0001) and alteration compared to the ref (45) value were observed for the groups that received subcutaneous magnet implantation (Figure 5a), indicating a possible correlation between magnet implantation and erythrocytopenia, like what was observed with chemotherapy.

A significant result was also observed in the determination of hemoglobin (Figure 5b). Even though there was a significant difference (with *p* 0.0003 in the group with magnet implantation, *p* 0.0024 in the group with magnet implantation and SPIONS, *p* 0.0121 in the group with magnet implantation and SPIONS and LASSBio-1735, and *p* < 0.0001 in the group treated with SPIONS and LASSBio-1735), this does not indicate a probable anemic condition. This is because we observed increased hemoglobin, a vital protein found within red blood cells and plasma, whose primary function is transporting oxygen. This suggests that even iron oxide SPIONS would not disrupt homeostasis, indicating its use for several therapeutic purposes.^46^

Continuing the evaluation, no significant alterations were found in the hematocrit analysis and platelet count, demonstrating that the compounds do not interfere with hemostasis.^47^

Several studies analyze the use of compounds with SPIONS for immunological and immunogenic therapy options, as currently used ones exhibit a carrier characteristic. Therefore, we evaluated total leukocytes (Figure 5e) from a complete blood count. In this analysis, we observed that almost all treatments analyzed are promising for therapy use. They do not exhibit toxicity or cause any alterations compared to reference values of cells whose primary function is defense in the body,^48,49^ and they do not cause morphological cellular alterations, as demonstrated in blood smears.

These alterations are observed in groups treated with magnet implantation and SPIONS (*p* 0.0089) and with doxorubicin (*p* 0.0115) (Figure 5c); however, for the semiacute toxicity test, leukopenic action was observed as an action already found in other chemotherapeutic agents.

Neutrophils and lymphocytes are fundamental to the inflammatory process. The neutrophil-to-lymphocyte ratio (NLR) is calculated by dividing the absolute neutrophil count by the lymphocyte count obtained from a complete peripheral blood count. The NLR is cheap and easy to obtain clinical data that indicates the inflammatory process in various other clinical conditions.^49^

The importance of the NLR in diagnosing and monitoring the evolution of neoplasms is being widely studied, given its importance and great applicability in the prognosis and progression of various neoplasms.^50,51^ However, no significant difference was found in our analyses, indicating that the treatments did not increase the inflammatory process, intensifying the neoplastic process in the analyzed animals.

The serum iron test aims to verify the concentration of iron in the blood, allowing the identification of whether there is a deficiency or overload of this mineral. Depending on the amount of iron in the blood, this can indicate nutritional deficiencies, anemia, or liver problems.^52−54^

Considering the composition of the SPIONS used in this study, assessing a possible increase in iron in the bloodstream may be related to potential intoxication, which can lead to various disorders accompanied by circulatory^53^ collapse. Fortunately, we did not observe this when evaluating the groups treated for 7 days.

A significant result was observed in the analysis of ALT, an enzyme considered the gold standard for diagnosing liver diseases because it is found only in the liver. No significant difference was found in the analyzed samples. The same was observed in the analysis of AST, indicating the absence of hepatotoxicity during the 7 days of treatment.

Some drugs commonly used to treat neoplastic processes have as one of their main side effects damage to the kidneys; for this reason, we evaluated the renal markers creatinine and urea. We found this possible renal reaction only in the group that received treatment with Doxorubicin, which showed a considerable increase that led to a significant difference in creatinine assessment (*p* 0.0311) (Figure 5d). An important difference was also observed in urea assessment, but this time with a reduction in urea for the groups that received Doxorubicin (*p* 0.0001) and the group that received the NP solution with LASSBio-1735 (*p* 0.0001) (Figure 5e).

Several tumors cause a significant reduction in appetite. The patient does not feel hungry or like eating. Similarly, some types of cancer cause nutritional depletion by themselves.^55−57^ Cancer treatment also has side effects that temporarily affect the patient, making it difficult for them to eat. Among the most frequent are nausea, vomiting, diarrhea, mucositis, and lack of appetite. These factors contribute to progressive weight loss during treatment, which can lead the patient to exaggerated states of malnutrition, known as cachexia.^28−30^

To assess possible cachexia, the variation in the animals’ weight at the beginning and end of treatment was evaluated, weighing on the first day and, at the end, weighing on the seventh day. No significant difference was observed, indicating that the treatments used did not cause relevant changes in weight.

In evaluating tumor weights, it was not possible to establish a correlation between treatments and weights, as a lower weight was observed in the control group compared to the group treated with doxorubicin, a chemotherapy drug with a known outcome. However, analyzing the results that show significant differences, it was demonstrated that the groups with magnet implantation and SPIONS (*p* 0.0026) and magnet implantation and SPIONS (*p* 0.0390) with the molecule presented the highest tumor weights (Figure 5f).

Furthermore, in the morphological evaluation of blood smears, no significant alterations were identified in the analyzed cells (data not shown). This analysis included a detailed observation of the morphology of erythrocytes, leukocytes, and platelets, confirming cellular integrity, the absence of deformities or irregularities, and the preservation of typical characteristics for each cell type. These findings support the hypothesis that the evaluated treatments, including subcutaneous magnet implantation, SPIONS, and their combination with LASSBio-1735, do not compromise the structure of blood cells, highlighting the safety of these procedures within the studied context.

When animal experimentation is deemed the only viable option, the number of laboratory animals utilized should be strictly limited to the minimum required to generate meaningful data. Moreover, comprehensive refinement measures must be implemented to minimize any pain, suffering, or distress resulting from the experimental procedures.

In this study, animal welfare was evaluated by assessing the conditions of their environment and considering critical aspects of their anatomy, physiology, ethology, and species-specific management. The analysis revealed no significant deviations in welfare scores across all groups studied (Table 2), suggesting that the treatments and management practices maintained the physical and mental balance of the animals within their environment over the 7 day period.

**Table 2:** Animal Welfare Assessment with Treatments over 7 Days

parameters	control	SPIONS	LASSBio-1735	subcutaneous magnet implant	subcutaneous magnet implantation and SPIONS	subcutaneous magnet implantation and SPIONS with LASSBio-1735	SPIONS with LASSBio-1735	doxorubicin
ulcer	0	0	0	0	0	0	0	0
coat	0	0	0	0	0	0	0	0
movement	0	0	0	0	0	0	0	0
posture	0	0	0	0	0	0	0	0
tail	0	0	0	0	0	0	0	0
eyes	0	0	0	0	0	0	0	0
ears	0	0	0	0	0	0	0	0
whiskers	0	0	0	0	0	0	0	0
end score	0	0	0	0	0	0	0	0

### Analysis of Results from Mice Treated for 14 Days

**Figure 6:**
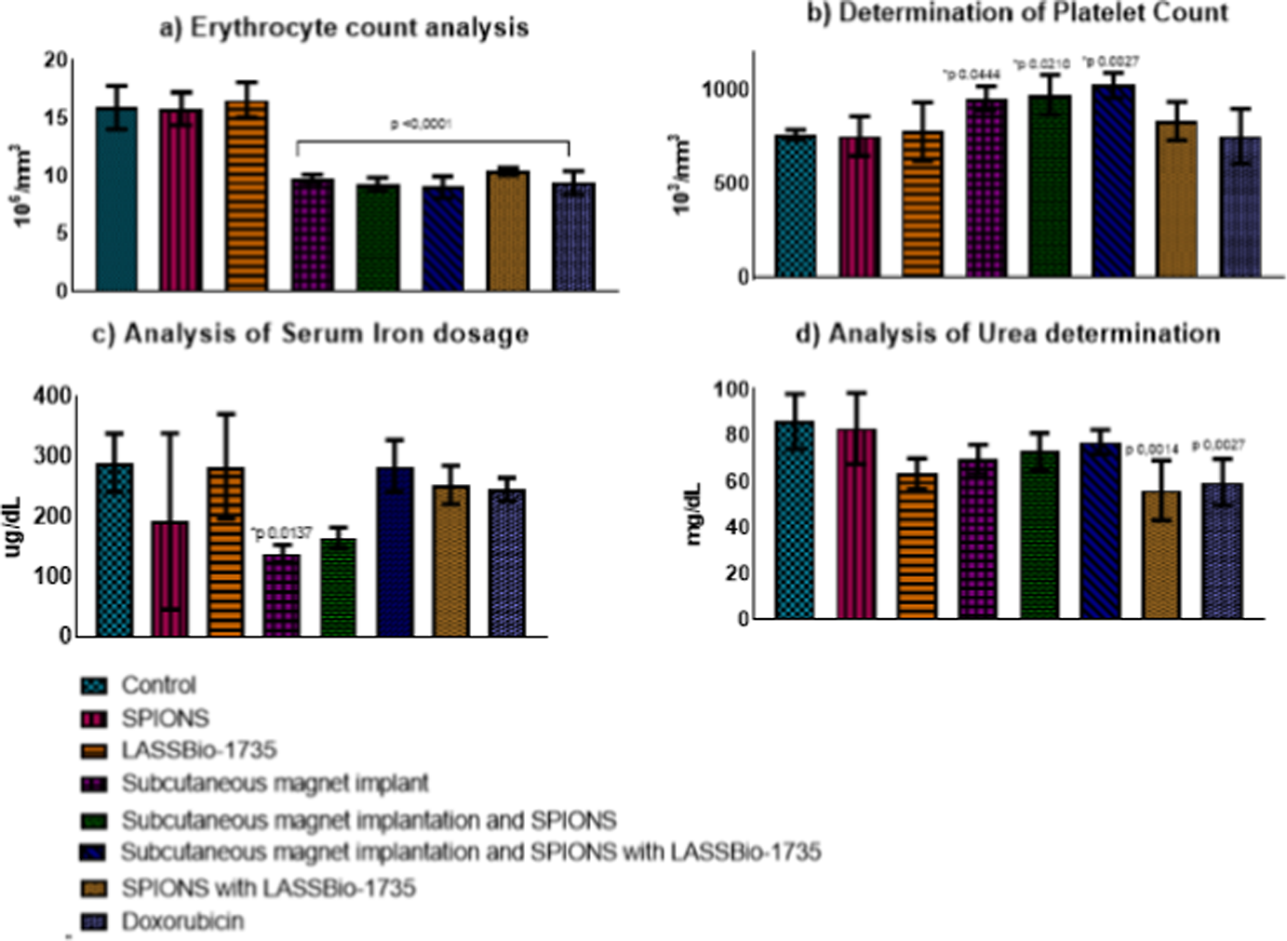
Myelotoxicity analysis, with an evaluation of red blood cells (a), platelet (b), and serum iron (c) and urea(d) over 14 days *p < 0.05.

In the assessment of subchronic toxicity, this phase provides an opportunity to refine the understanding of dose–response relationships following repeated administrations while enabling detailed comparisons between toxicity studies. Consistent changes were observed during this intermediate evaluation, with statistically significant differences (*p* < 0.0001) in red blood cell counts (Figure 6a) for the same treatment groups when compared to the 7 day analysis period. This observation may be linked to the progression of tumor mass, which potentially results in hemorrhagic lesions that influence red blood cell levels.

Additionally, this possible hemorrhagic effect could account for the increase in platelet counts observed (Figure 6b). Significant differences were recorded in the group subjected to the magnet implant (*p* = 0.0444), as well as in those receiving the magnet implant alongside SPIONS (*p* = 0.0210), and the combination of the magnet implant, SPIONS, and the molecule (*p* = 0.0027). These outcomes suggest a physiological adaptation aimed at enhancing platelet production to mitigate potential hemorrhagic challenges.

The changes observed in the serum analyses are consistent with the morphological analysis observed in the blood smears, which did not show morphological alterations as depicted in.

No significant changes were found in the total leukocyte investigation and in the neutrophil/lymphocyte ratio, recently popularized as a biomarker of systemic inflammatory response, which proves to be an excellent analysis in neoplastic studies and corroborates the survival of those treated in our study.

The significant alteration found in the treatment with a magnet implant (p 0.0137) (Figure 6c) suggests that the decrease in circulating serum iron is likely related to the magnet’s magnetic action, attracting the serum iron to the vicinity of the application. However, confirming this action is only possible with histopathological analysis, with iron deposits near the tumor masses.

Prolonged treatments tend to cause liver damage; thus, this subchronic test indicates that even though our treatments are metabolized by the liver, they do not induce hepatic toxicity,^58^ as demonstrated in the analysis of liver enzymes, such as ALT, which is typically elevated in the presence of liver lesions caused by toxic drugs or infections, and AST in cases where hepatocyte integrity may be compromised due to necrosis or inflammation.^59^

In the evaluation of serum creatinine, no significant alteration was found in the assessed treatments. However, in the urea analysis (Figure 6d), alterations were found, with reductions in the groups that received SPIONS associated with LASSBio-1735 (*p* 0.0014) and doxorubicin (*p* 0.0027). Even though urea is not a gold-standard marker for possible nephropathy, these findings are of the utmost importance, as they allow comparison with the results of a known drug since nephrotoxicity is one of the main harmful effects caused by the administration of doxorubicin.^60^

Due to the kidneys’ low regenerative capacity, they are more susceptible to cytotoxic damage. Epithelial injuries are the first to appear, leading to degeneration of the glomeruli. The final course of this is glomerulosclerosis, the main nephropathy related to the use of doxorubicin.^61,62^

The normal and consistent variations observed over the 14 days were insignificant, suggesting that the treatments enhanced protein metabolism. Consequently, the body requires more calories to perform its daily functions, which can lead to weight loss—a common symptom in neoplasms. The differences in tumor masses are noticeable, but to assess slight variations, one should consider differences in tumor growth rates and the lack of significant differences observed in this evaluation.

Unfortunately, there is limited literature on animal welfare analysis specifically for doxorubicin, as it was the only treatment that registered above a score of 0 over these 14 days for further discussion. Therefore, it can be concluded that, based on our research group’s planned treatments and the results mentioned above, the 14 day treatment period did not reveal any significant toxicity or issues related to the subcutaneous implantation of magnets in the animals. Table 3 shows animal welfare assessment with treatments over 14 days.

**Table 3:** Animal Welfare Assessment with Treatments over 14 Days

parameters	control	SPIONS	LASSBio-1735	subcutaneous magnet implant	subcutaneous magnet implantation and SPIONS	subcutaneous magnet implantation and SPIONS with LASSBio-1735	SPIONS with LASSBio-1735	doxorubicin
ulcer	0	0	0	0	0	0	0	0
coat	0	0	0	0	0	0	0	1
movement	0	0	0	0	0	0	0	2
posture	0	0	0	0	0	0	0	1
tail	0	0	0	0	0	0	0	1
eyes	0	0	0	0	0	0	0	0
ears	0	0	0	0	0	0	0	0
whiskers	0	0	0	0	0	0	0	0
end score	0	0	0	0	0	0	0	5

### Analysis of Results from Mice Treated for 30 Days

**Figure 7:**
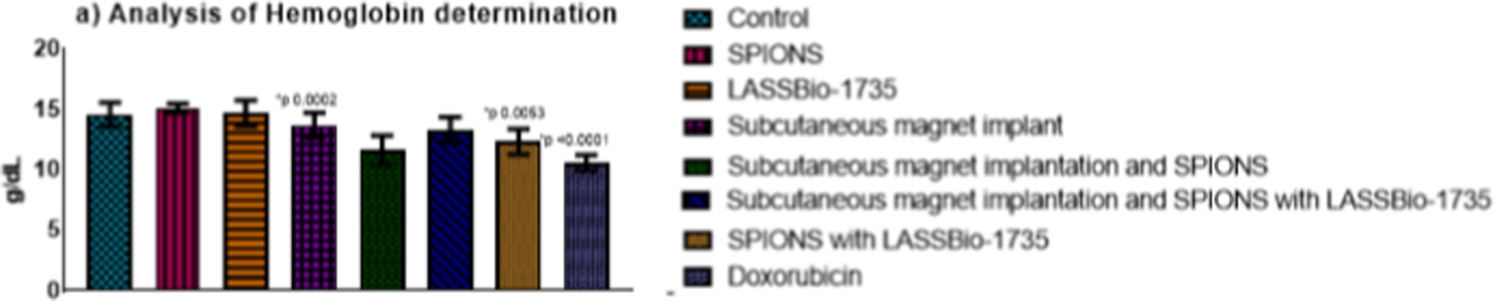
Myelotoxicity analysis, with evaluation hemoglobin, over 30 days, *p < 0.05.

In the chronic toxicity evaluation, significant alterations were identified (Figure 7) in the group that received SPIONS associated with LASSBio-1735 (*p* = 0.002), the group with subcutaneous magnet implantation and SPIONS associated with LASSBio-1735 (*p* = 0.0053), and the group treated with doxorubicin (*p* < 0.0001).^8^ These alterations were accompanied by variations in hemoglobin levels, suggesting a direct correlation between the observed changes and the organism’s adaptation to the treatment. Despite these alterations, the values remained within the reference ranges,^60^ indicating that the treatments do not interfere with hemostasis or the characterization of anemia due to drug toxicity from prolonged use.^46,47^

A more detailed analysis of red blood cell counts over the three evaluated periods showed an initial reduction in some groups, possibly related to adaptation to tumor growth or the initial impact of the treatments. However, this trend was not sustained over the longer analysis period, suggesting that the organism was able to reestablish hematopoietic balance. This recovery is evidenced by the normalization of hemoglobin and platelet counts, reinforcing the organism’s physiological compensatory capacity.

Additionally, no significant changes were observed in cell morphology or the leukogram. The analysis of leukocytes revealed that lymphocytes, which account for approximately 75% of the total, remain predominant in mice, followed by neutrophils.^63^ The absence of alterations in lymphocyte and neutrophil counts indicates that intense inflammatory processes or exacerbated immune responses did not occur during the 30 day period, unlike what is typically observed in advanced neoplastic conditions.

No changes were detected in serum iron concentration, which can be attributed to the restoration of hemostasis. This excludes iron-deficiency anemia as a possible complication, reinforcing the treatments’ ability to avoid severe adverse effects. However, histological analyses are ongoing to assess potential iron deposits in the tissues.

Based on the renal function results and the data collected over 30 days, it is presumed that the tested treatments are safe and do not present significant toxicity. However, despite the observed safety, it has not yet been possible to confirm an antineoplastic action in the evaluated groups. Significant differences indicating a potential antineoplastic effect of the treatments were not evidenced, and confirmation of this hypothesis will depend on the ongoing biomolecular results.

In the well-being assessment, we observed relevant alterations, as around the 20th day, the onset of redness points was observed, which progressed to ulcers that quickly healed near the sites of magnet application in almost all groups.

The most significant changes were observed in the control group and the group that received doxorubicin, with scores of 8 and 10, values much higher than those found in the other studied groups. This may indicate that the treatments could have had a protective effect on the animals, which could be further elucidated by comparing all periods and analyses, including gene expression of inflammatory markers and oxidative stress and fatigue, to be conducted later (Table 4).

**Table 4:** Animal Welfare Assessment with Treatments over 30 Days

parameters	control	SPIONS	LASSBio-1735	subcutaneous magnet implant	subcutaneous magnet implantation and SPIONS	subcutaneous magnet implantation and SPIONS with LASSBio-1735	SPIONS with LASSBio-1735	doxorubicin
ulcer	5	3	3	3	3	4	4	3
coat	1	1	0	2	0	0	0	4
movement	0	0	0	0	0	0	0	0
posture	0	0	0	0	0	0	0	1
tail	1	0	0	0	0	0	0	1
eyes	0	0	0	0	0	0	0	0
ears	0	0	0	0	0	0	0	0
whiskers	0	0	0	0	0	0	0	0
end score	8	4	3	5	3	3	4	10

## Conclusion

In conclusion, to evaluate the toxicity of the compounds and the effectiveness of subcutaneous magnet implantation, it was possible to observe that both the isolated compounds, such as SPIONS and LASSBio-1735, and their combinations, despite yielding different results in the analyzed periods, ensured the survival and well-being of the animals analyzed. Besides, nonrelevant toxicity was found in the studied groups. This opens further research possibilities, including exploring different cellular lineages in future stages.

## Abbreviations

ALT: alanine aminotransferase
AST: aspartate aminotransferase
CA-4: combretastatin A4
EAC: Ehrlich ascites carcinoma
EDTA: ethylenediaminetetraacetic acid
FTIR: Fourier-transform infrared spectroscopy
IP: intraperitoneal injection
LASSBio: Laboratory for Evaluation and Synthesis of Bioactive Substances
NLR: neutrophil-to-lymphocyte ratio NLR
SPIONs: superparamagnetic iron oxide nanoparticles
SQUID: superconducting quantum interference device
WHO: World Health Organization
XRPD: X-ray powder diffraction.

## References

[ref1] Santos M. D. O.; Lima F. C. D. S. D.; Martins L. F. L.; Oliveira J. F. P.; Almeida L. M. d.; Cancela M. d. C. Estimativa de Incidência de Câncer no Brasil, 2023-2025. Rev. Bras Cancerol 2023, 69, e213700 10.32635/2176-9745.RBC.2023v69n1.3700.

[ref2] Miranda G. M. D.; Mendes A. D. C. G.; Silva A. L. A. D. Population aging in Brazil: current and future social challenges and consequences. Rev. bras geriatr gerontol 2016, 19, 507–519. 10.1590/1809-98232016019.150140.

[ref3] Fonseca Travassos G.; Bragança Coelho A.; Arends-Kuenning M. P. The elderly in Brazil: demographic transition, profile, and socioeconomic condition. Rev. bras estud popul 2020, 37, 1–27. 10.20947/S0102-3098a0129.

[ref4] INCA Estimativa 2023: incidência de câncer no Brasil; Instituto Nacional De Câncer: Rio de Janeiro, RJ, 2023

[ref5] Brown J. S.; Amend S. R.; Austin R. H.; et al. Updating the Definition of Cancer. Mol. Cancer Res. 2023, 21, 1142–1147. 10.1158/1541-7786.MCR-23-0411. 37409952 PMC10618731

[ref6] Gavas S.; Quazi S.; Karpiński T. M. Nanoparticles for Cancer Therapy: Current Progress and Challenges. Nanoscale Res. Lett. 2021, 16, 173 10.1186/s11671-021-03628-6. 34866166 PMC8645667

[ref7] Chen D.; Milacic V.; Frezza M.; Dou Q. Metal Complexes, their Cellular Targets and Potential for Cancer Therapy. CPD 2009, 15, 777–791. 10.2174/138161209787582183. 19275642

[ref8] Orvig C.; Abrams M. J. Medicinal Inorganic Chemistry: Introduction. Chem. Rev. 1999, 99, 2201–2204. 10.1021/cr980419w. 11749478

[ref9] Benite A. M. C.; Machado S. d. P.; Barreiro E. J. Considerações sobre a Química Bioinorgânica Medicinal. Rev. Eletr Farm 2007, 4, 131 10.5216/ref.v4i2.3027.

[ref10] Mjos K. D.; Orvig C. Metallodrugs in Medicinal Inorganic Chemistry. Chem. Rev. 2014, 114, 4540–4563. 10.1021/cr400460s. 24456146

[ref11] Guo Z.; Sadler P. J. Metals in Medicine. Angew. Chem. Int. Ed 1999, 38, 1512–1531. 10.1002/(SICI)1521-3773(19990601)38:11<1512::AID-ANIE1512>3.0.CO;2-Y. 29711002

[ref12] Rossi L. M.; Costa N. J. S.; Silva F. P.; Wojcieszak R. Magnetic nanomaterials in catalysis: advanced catalysts for magnetic separation and beyond. Green Chem. 2014, 16, 2906 10.1039/c4gc00164h.

[ref13] Long Y.; Xie M.; Niu J.; et al. Preparation of acid–base bifunctional core–shell structured Fe3O4@SiO2 nanoparticles and their cooperative catalytic activity. Appl. Surf. Sci. 2013, 277, 288–292. 10.1016/j.apsusc.2013.04.050.

[ref14] Hussein M. Z.; Al Ali S.; Geilich B.; et al. Synthesis, characterization, and antimicrobial activity of an ampicillin-conjugated magnetic nanoantibiotic for medical applications. IJN 2014, 3801, 3801 10.2147/IJN.S61143. PMC4134181 25143729

[ref15] Lei W.; Min W.; Hui D.; et al. Effect of Surface Modification on Cellular Internalization of Fe_3_O_4_ Nanoparticles in Strong Static Magnetic Field. J. Nanosci. Nanotechnol. 2015, 15, 5184–5192. 10.1166/jnn.2015.9841. 26373103

[ref16] Nesztor D.; Bali K.; Tóth I. Y.; et al. Controlled clustering of carboxylated SPIONs through polyethylenimine. J. Magn. Magn. Mater. 2015, 380, 144–149. 10.1016/j.jmmm.2014.10.091.

[ref17] Zhang X. F.; Mansouri S.; Mbeh D. A.; et al. Nitric Oxide Delivery by Core/Shell Superparamagnetic Nanoparticle Vehicles with Enhanced Biocompatibility. Langmuir 2012, 28, 12879–12885. 10.1021/la302357h. 22892047

[ref18] Lima R. D.; Oliveira J. L.; Murakami P. S. K.; et al. Iron oxide nanoparticles show no toxicity in the comet assay in lymphocytes: A promising vehicle as a nitric oxide releasing nanocarrier in biomedical applications. J. Phys.: Conf. Ser. 2013, 429, 012021 10.1088/1742-6596/429/1/012021.

[ref19] Haddad P. S., Seabra A. B. Biomedical applications of magnetic nanoparticles. In: Iron Oxides: Structure, Properties and Applications, 1st ed. Nova Science Publishers, Inc., New York, USA, 2012; pp 165–188.

[ref20] Losito D. W.; de Araujo D. R.; Bezzon V. D. N.; et al. Mesoporous Silica-Fe3O4 Nanoparticle Composites as Potential Drug Carriers. ACS Appl. Nano Mater. 2021, 4, 13363–13378. 10.1021/acsanm.1c02861.

[ref21] Britos T. N.; Castro C. E.; Bertassoli B. M.; et al. In vivo evaluation of thiol-functionalized superparamagnetic iron oxide nanoparticles. Mater. Sci. Eng., C 2019, 99, 171–179. 10.1016/j.msec.2019.01.118. 30889689

[ref22] Ravelli R. B. G.; Gigant B.; Curmi P. A.; et al. Insight into tubulin regulation from a complex with colchicine and a stathmin-like domain. Nature 2004, 428, 198–202. 10.1038/nature02393. 15014504

[ref23] Silva J. C.; Oliveira Júnior R. G. d.; Silva M. G. e.; Lavor É. M. d.; Soares J. M. D.; Lima-Saraiva S. R. G. d.; Diniz T. C.; Mendes R. L.; Alencar Filho E. B. d.; Barreiro E. J. d. L.; et al. LASSBio-1586, an N-acylhydrazone derivative, attenuates nociceptive behavior and the inflammatory response in mice. PLoS One 2018, 13, e0199009 10.1371/journal.pone.0199009. 30059558 PMC6066216

[ref24] De Figueiredo L. P.; Ibiapino A. L.; Do Amaral D. N.; et al. Structural characterization and cytotoxicity studies of different forms of a combretastatin A4 analogue. J. Mol. Struct. 2017, 1147, 226–234. 10.1016/j.molstruc.2017.06.093.

[ref25] Ehrlich P. Experimentelle Karzinomstudien an Mäusen. Z. Aerztl. Fortbild. 1906, 3, 205–213.

[ref26] Fernandes P. D.; Guerra F. S.; Sales N. M.; et al. Characterization of the inflammatory response during Ehrlich ascitic tumor development. J. Pharmacol. Toxicol. Methods 2015, 71, 83–89. 10.1016/j.vascn.2014.09.001. 25199596

[ref27] Bruijnincx P. C.; Sadler P. J. New trends for metal complexes with anticancer activity. Curr. Opin. Chem. Biol. 2008, 12, 197–206. 10.1016/j.cbpa.2007.11.013. 18155674 PMC2923029

[ref28] Felipe K. B.; Kviecinski M. R.; Da Silva F. O.; et al. Inhibition of tumor proliferation associated with cell cycle arrest caused by extract and fraction from Casearia sylvestris (Salicaceae). J. Ethnopharmacol. 2014, 155, 1492–1499. 10.1016/j.jep.2014.07.040. 25077466

[ref29] Abdel-Rahman M. N.; Kabel A. M. Comparative study between the effect of methotrexate and valproic acid on solid Ehrlich tumour. Journal of the Egyptian National Cancer Institute 2012, 24, 161–167. 10.1016/j.jnci.2012.08.001. 23159286

[ref30] Salgado Oloris S. C.; Dagli M. L. Z.; Guerra J. L. Effect of β-carotene on the development of the solid Ehrlich tumor in mice. Life Sci. 2002, 71, 717–724. 10.1016/S0024-3205(02)01730-7. 12072159

[ref31] Loewenthal H.; Jahn G. Übertragunsversuche mit carcinomatöser Mäuse-Ascitesflüssigkeit und ihr Verhalten gegen physikalische und chemische Einwirkungen. Z Krebs-forsch 1932, 37, 439–447. 10.1007/BF01618550.

[ref32] Glover T., Mitchell K. An Introduction to Biostatistics, 3rd ed.; Waveland Press, Inc, 2015

[ref33] Singh J. The National Centre for the Replacement, Refinement, and Reduction of Animals in Research. J. Pharmacol. Pharmacother. 2012, 3, 87–89. 10.1177/0976500X20120105. 22368436 PMC3284057

[ref34] Molina M. M.; Seabra A. B.; De Oliveira M. G.; et al. Nitric oxide donor superparamagnetic iron oxide nanoparticles. Mater. Sci. Eng. C 2013, 33, 746–751. 10.1016/j.msec.2012.10.027. 25427482

[ref35] Bish D. L.; Howard S. A. Quantitative phase analysis using the Rietveld method. J. Appl. Crystallogr. 1988, 21, 86–91. 10.1107/S0021889887009415.

[ref36] Hill R. J.; Howard C. J. Quantitative phase-analysis from neutron powder diffraction data using the Rietveld method. J. Appl. Crystallogr. 1987, 20, 467–474. 10.1107/S0021889887086199.

[ref37] Rietveld H. M. A profile refinement method for nuclear and magnetic structures. J. Appl. Crystallogr. 1969, 2, 65–71. 10.1107/S0021889869006558.

[ref38] Fleet M. E. The structure of magnetite. Acta Crystallographica Section B Structural Crystallography and Crystal Chemistry 1981, 37, 917–920. 10.1107/S0567740881004597.

[ref39] Moggach S. A.; Allan D. R.; Parsons S.; et al. The effect of pressure on the crystal structure of hexagonal L-cystine. J. Synchrotron Radiat. 2005, 12, 598–607. 10.1107/S0909049505019850. 16120983

[ref40] Rowles M. R. pdCIFplotter: visualizing powder diffraction data in pdCIF format. J. Appl. Crystallogr. 2022, 55, 631–637. 10.1107/S1600576722003478. 35719308 PMC9172038

[ref41] Larsen E. K.; Nielsen T.; Wittenborn T.; et al. Size-Dependent Accumulation of PEGylated Silane-Coated Magnetic Iron Oxide Nanoparticles in Murine Tumors. ACS Nano 2009, 3, 1947–1951. 10.1021/nn900330m. 19572620

[ref42] Ramachandran E.; Natarajan S. Crystal growth of some urinary stone constituents: III. In-vitro crystallization of L-cystine and its characterization. Cryst. Res. Technol. 2004, 39, 308–312. 10.1002/crat.200310187.

[ref43] Pandey C. M.; Sumana G.; Malhotra B. D. Microstructured Cystine Dendrites-Based Impedimetric Sensor for Nucleic Acid Detection. Biomacromolecules 2011, 12, 2925–2932. 10.1021/bm200490b. 21650182

[ref44] Girija E. K.; Kalkura S. N.; Ramasamy P. Crystallization of cystine. J. Mater. Sci.: Mater. Med. 1995, 6, 617–619. 10.1007/BF00123439.

[ref45] Pessini P. G. D. S.; Knox De Souza P. R.; Chagas C. D. S.; Sampaio E. G.; Neves D. S.; Petri G.; Fonseca F. L. A.; da Silva E. B. Hematological reference values and animal welfare parameters of BALB/C-FMABC (*Mus musculus*) inoculated with Ehrlich tumor kept in the vivarium at ABC Medical School. Anim Models and Exp Med. 2020, 3, 32–39. 10.1002/ame2.12099. PMC7167238 32318657

[ref46] Karazawa E. H. I.; Jamra M. Parâmetros hematológicos normais. Rev. Saúde Pública 1989, 23, 58–66. 10.1590/S0034-89101989000100008. 2683016

[ref47] de Benoist B.; McLean E.; Egli I.; Cogswell M. Worldwide prevalence of anaemia 1993–2005: WHO global database on anaemia; World Health Organization, 2008 10.1017/S1368980008002401 18498676

[ref48] Naoum F. A., Naoum P. C. Hematologia laboratorial. Leucócitos, 1st ed. Ed.a Academia de Ciência e Tecnologia, São José do Rio Preto, SP, 2006

[ref49] Guthrie G. J. K.; Charles K. A.; Roxburgh C. S. D.; et al. The systemic inflammation-based neutrophil–lymphocyte ratio: Experience in patients with cancer. Critical Reviews in Oncology/Hematology 2013, 88, 218–230. 10.1016/j.critrevonc.2013.03.010. 23602134

[ref50] Teramukai S.; Kitano T.; Kishida Y.; et al. Pretreatment neutrophil count as an independent prognostic factor in advanced non-small-cell lung cancer: An analysis of Japan Multinational Trial Organisation LC00–03. Eur. J. Cancer 2009, 45, 1950–1958. 10.1016/j.ejca.2009.01.023. 19231158

[ref51] Szarfarc S. C. Políticas públicas para o controle da anemia ferropriva. Rev. Bras Hematol Hemoter 2010, 32, 02 10.1590/S1516-84842010005000065.

[ref52] Hershko C.; Camaschella C. How I treat unexplained refractory iron deficiency anemia. Blood 2014, 123, 326–333. 10.1182/blood-2013-10-512624. 24215034

[ref53] Okam M. M.; Koch T. A.; Tran M.-H. Iron Supplementation, Response in Iron-Deficiency Anemia: Analysis of Five Trials. Am. J. Med. 2017, 130, 991e1–991e8. 10.1016/j.amjmed.2017.03.045. 28454902

[ref54] Aithal G. P.; Rawlins M. D.; Day C. P. Clinical diagnostic scale: a useful tool in the evaluation of suspected hepatotoxic adverse drug reactions. Journal of Hepatology 2000, 33, 949–952. 10.1016/S0168-8278(00)80127-0. 11131457

[ref55] Biesalski H.-K.; Dragsted L. O.; Elmadfa I.; et al. Bioactive compounds: Definition and assessment of activity. Nutrition 2009, 25, 1202–1205. 10.1016/j.nut.2009.04.023. 19695833

[ref56] Bonilla L.; Ben-Aharon I.; Vidal L.; et al. Dose-Dense Chemotherapy in Nonmetastatic Breast Cancer: A Systematic Review and Meta-analysis of Randomized Controlled Trials. JNCI Journal of the National Cancer Institute 2010, 102, 1845–1854. 10.1093/jnci/djq409. 21098761 PMC3001963

[ref57] Bossola M.; Pacelli F.; Rosa F.; et al. Does Nutrition Support Stimulate Tumor Growth in Humans?. Nut in Clin Prac 2011, 26, 174–180. 10.1177/0884533611399771. 21447771

[ref58] Nelson D. L., Cox M. M. Lehninger Principles of Biochemistry, 7th ed.; W. H. Freeman, 2017

[ref59] Reis E. ´. G. d. Ensaio cl’inico comparativo entre Itraconazol e associação de Itraconazol e iodeto de potássio no tratamento da esporotricose felina. Ph.D. Thesis, Fundação Oswaldo Cruz, 2016.

[ref60] Hortobágyi G. N. Anthrazykline in der Krebstherapie: Ein Überblick. Drugs 1997, 54, 1–7. 10.2165/00003495-199700544-00003.

[ref61] Rook M.; Lely A. T.; Kramer A. B.; et al. Individual differences in renal ACE activity in healthy rats predict susceptibility to adriamycin-induced renal damage. Nephrol., Dial., Transplant. 2005, 20, 59–64. 10.1093/ndt/gfh579. 15572383

[ref62] Birben E.; Sahiner U. M.; Sackesen C.; et al. Oxidative Stress and Antioxidant Defense. World Allergy Organ. J. 2012, 5, 9–19. 10.1097/WOX.0b013e3182439613. 23268465 PMC3488923

[ref63] Eeverds N. Hematology of the Laboratory Mouse. In The Mouse in Biomedical Research; Elsevier, 2007; pp 133–170.

